# The Impact of Ustekinumab Biosimilar on Therapeutic Strategies in Crohn's Disease: A Paradigm Shift?

**DOI:** 10.1002/ueg2.12717

**Published:** 2024-11-30

**Authors:** C. Bezzio, A. Armuzzi

**Affiliations:** ^1^ IBD Unit Gastroenterology Department IRCCS Humanitas Research Hospital Rozzano Italy; ^2^ Department of Biomedical Sciences Humanitas University Pieve Emanuele Italy

**Keywords:** anti‐IL‐23, anti‐IL12/23, biosimilars, costs, earlier treatment, IBD, inflammatory bowel disease, risankizumab, saving costs, sustainability

The advent of Ustekinumab biosimilars marks a significant turning point in the treatment of Crohn's disease (CD) [1]. By making advanced treatments more accessible, biosimilars could enable a shift toward earlier intervention, potentially improving long‐term outcomes and supporting sustainable healthcare strategies. Therefore, after the entry of anti‐TNF alpha biosimilars, cost reductions associated with Ustekinumab biosimilars can further transform approaches to CD management [2].

## 1 Revolutionizing Crohn's Disease Treatment With Biosimilars

Recent studies reinforce the efficacy of an “early”/“top‐down” approach in active CD, which demonstrates better long‐term outcomes than the traditional “step‐up” strategy: an early, aggressive treatment strategy is therefore essential to prevent disease progression in CD [3].

The advent of anti‐TNF alpha biosimilars in the last years has reshaped inflammatory bowel disease (IBD) therapy, with Ustekinumab biosimilars poised to extend these changes. Ustekinumab has traditionally been essential for moderate‐to‐severe CD management [4], but its cost has restricted widespread use.

Anti‐TNF drugs have so far generally been used as first‐line treatment in patients with moderate to severe CD. However, the SEAVUE trial, which demonstrated comparable efficacy between Ustekinumab and Adalimumab in CD [5], provides a strong foundation for Ustekinumab adoption in patients naïve to biologic therapies.

Even more recently, the SEQUENCE study [6] was published, comparing the efficacy and safety of Risankizumab (the only currently approved anti‐IL‐23 for CD) versus Ustekinumab in patients with moderate‐to‐severe CD who had an inadequate response or adverse effects from prior anti‐TNF therapies. Results demonstrated that Risankizumab met noninferiority criteria for clinical remission at week 24 and showed superior efficacy in achieving endoscopic remission at week 48. Safety profiles were comparable between the two groups, supporting Risankizumab as a potentially more effective option in inducing endoscopic healing in this patient population.

Moreover, preliminary data on Guselkumab, an anti‐IL‐23, have recently been presented, indicating the superiority of this drug compared to Ustekinumab in both naïve patients and those previously exposed to anti‐TNF therapy [7].

Vedolizumab, an anti‐integrin alpha‐4 beta‐7 agent, has also demonstrated good efficacy in the treatment of moderate‐to‐severe CD, showing comparable efficacy to Ustekinumab in real‐world studies [8, 9].

## 2 The Role of Real‐World Evidence and Ongoing Challenges

A future challenge will be to determine the positioning of these drugs, balancing the excellent efficacy and safety profile of anti‐IL‐23 agents, which preliminary data suggest may be superior to Ustekinumab, with the known efficacy, safety, and affordable cost of Ustekinumab in inducing and maintaining remission in patients with moderate‐to‐severe CD.

Therefore, Ustekinumab biosimilars could be an ideal first‐line option, even in patients with some coexisting extraintestinal manifestations, such as psoriasis or psoriatic arthritis [3].

Despite the promise of Ustekinumab biosimilars, several challenges remain. Real‐world data on long‐term efficacy and safety are still limited, underscoring the need for more comprehensive studies.

## 3 Possible Barriers to the Use of Ustekinumab Biosimilars

A potential barrier to the use of Ustekinumab biosimilars could be the “nocebo effect,” where patient skepticism toward biosimilars may impact adherence and perceived efficacy [10]. Effective education and transparent communication about the safety and efficacy of biosimilars will be essential to overcome this barrier. Multidisciplinary care teams, including physicians, nurses, and pharmacists, can play a crucial role in addressing patient concerns and encouraging adherence [10].

## 4 Conclusions

The emergence of Ustekinumab biosimilars marks a significant advancement in the treatment of CD, enabling earlier intervention and greater accessibility due to reduced costs. The positive outcomes demonstrated in studies like SEAVUE reinforce the potential of Ustekinumab as first line therapy to effectively induce and maintain remission. Furthermore, ongoing research into anti‐IL‐23 agents such as Risankizumab and Guselkumab presents new options that may surpass Ustekinumab in certain contexts.

As the treatment landscape evolves, it will be crucial to address the challenges associated with patient adherence and perceptions of biosimilars. Continued investigation into the long‐term efficacy and safety of Ustekinumab biosimilars will be vital to establishing their role in CD management. Ultimately, a shift toward a more inclusive and cost‐effective treatment strategy can enhance patient outcomes and reduce disease‐related complications.

## Conflicts of Interest

CB served as consultant or received lecture fee from: AbbVie, Alfasigma, Celltrion, Eli Lilly, Ferring, Fresenius Kabi, Galapagos, Lionhealth, Johnson & Johnson, MSD, Pfizer, Takeda. AA served as consultant or received lecture fees from: Abbvie, Abivax, AG Pharma, Alfasigma, Astra Zeneca, Biogen, Boehringer Ingelheim, Bristol‐Myers Squibb, Celltrion, Eli‐Lilly, Ferring, Galapagos, Gilead, Giuliani, Janssen, Lionhealth, Merck, Nestlé, Pfizer, Protagonist Therapeutics, Roche, Sanofi, Samsung Bioepis, Sandoz, Takeda, Teva Pharmaceuticals, Tillots Pharma.
